# Daily Satellite Observations of Methane from Oil and Gas Production Regions in the United States

**DOI:** 10.1038/s41598-020-57678-4

**Published:** 2020-01-28

**Authors:** Joost A. de Gouw, J. Pepijn Veefkind, Esther Roosenbrand, Barbara Dix, John C. Lin, Jochen Landgraf, Pieternel F. Levelt

**Affiliations:** 1 Cooperative Institute for Research in Environmental Sciences, University of Colorado, Boulder, CO United States; 2 Department of Chemistry, University of Colorado, Boulder, CO United States; 3 Royal Netherlands Meteorological Institute, de Bilt, The Netherlands; 4 Faculty of Civil Engineering and Geosciences, Delft University of Technology, Delft, The Netherlands; 5 Department of Atmospheric Sciences, University of Utah, Salt Lake City, UT United States; 6 SRON Netherlands Institute for Space Research, Utrecht, The Netherlands

**Keywords:** Atmospheric chemistry, Atmospheric chemistry

## Abstract

Production of oil and natural gas in North America is at an all-time high due to the development and use of horizontal drilling and hydraulic fracturing. Methane emissions associated with this industrial activity are a concern because of the contribution to climate radiative forcing. We present new measurements from the space-based TROPOspheric Monitoring Instrument (TROPOMI) launched in 2017 that show methane enhancements over production regions in the United States. In the Uintah Basin in Utah, TROPOMI methane columns correlated with *in-situ* measurements, and the highest columns were observed over the deepest parts of the basin, consistent with the accumulation of emissions underneath inversions. In the Permian Basin in Texas and New Mexico, methane columns showed maxima over regions with the highest natural gas production and were correlated with nitrogen-dioxide columns at a ratio that is consistent with results from *in-situ* airborne measurements. The improved detail provided by TROPOMI will likely enable the timely monitoring from space of methane emissions associated with oil and natural gas production.

## Introduction

Methane is the second most important anthropogenic greenhouse gas in the atmosphere after carbon dioxide^1^. Methane emissions are related to (i) the production of fossil fuels, i.e. crude oil, natural gas and coal, and can also be released from natural seeps, (ii) microbial processes in wetlands, rice cultivation, livestock, landfills and termites, and (iii) biomass burning^2^. Because of the imbalance between methane sources and sinks, the atmospheric mixing ratio has increased from a pre-industrial value of 722 ppbv^1^ to the current global average of approximately 1850 ppbv^3^. As the atmospheric lifetime of methane is relatively short at 9.1 years^1^, a reduction in methane emissions would lower the combined radiative forcing from greenhouse gases on a timescale of years and is therefore a relatively efficient option to mitigate climate change.

Methane emissions associated with the production of fossil fuels (coal, oil and natural gas) constitute about 24% of the global emissions of methane and a larger fraction of the anthropogenic emissions. The global emissions from this industry have stayed relatively constant since 1985 even as the global production of fossil fuels increased over this same time period^2^. Methane emissions from the U.S. oil and natural gas supply chain have received much recent attention and are estimated to be 2.3% of gross U.S. gas production^4^ and 41% of the anthropogenic U.S. emissions^5^. The long-term trends in U.S. emissions of methane are the subject of some debate, with one study finding a 30% increase between 2002–2014^6^, and another finding no significant trends between 2006–2015^7^.

Global measurements of methane are made from a network of ground-based and airborne *in-situ* measurements^8^. These measurements are mostly made away from sources to reflect background concentrations. Measurements of methane emissions in oil and gas production areas have been made using surface monitors^9,10^ and from research aircraft^11–13^. The latter are labor intensive and only provide snapshots of emissions, which may be an issue as episodic, large emissions can represent a relatively large fraction of the total^14^. This calls for more frequent, basin-wide monitoring of emissions, which explains the interest in methane measurements using space-based sensors. However, observing methane from oil and gas operations from space has been challenging. Schneising *et al*. derived methane emissions from two oil producing regions, the Bakken and Eagle Ford, using data from the SCIAMACHY instrument^15^. Kort *et al*. described the observation in SCIAMACHY data of a hotspot in methane over the Four Corners region, a region with natural gas and coal-bed methane production^16^. Turner *et al*. used data from the GOSAT instrument to describe trends in U.S. methane emissions but could not attribute the trends to a specific sector^6^. All of these studies presented satellite measurements of methane that were averaged over a year or more. One study that used observations from single satellite overpasses was reported by Thompson *et al*. following the Aliso Canyon blow-out^17^, which was one of the largest accidental releases of methane in recent history^18^. Similarly large emissions of methane in Turkmenistan were recently reported using data from GHGSat and TROPOMI^19^.

The TROPOspheric Monitoring Instrument (TROPOMI) is the sole instrument on the EU Copernicus Sentinel 5 Precursor satellite that was launched in October 2017. In comparison with its predecessor, the Ozone Monitoring Instrument (OMI)^20^, TROPOMI has an improved spatial resolution of up to 7 × 3.5 km^2^ and also added a short-wave infrared radiation (SWIR) band that allows the detection of methane and carbon monoxide^21^. The TROPOMI measurements of methane have been compared with those from the GOSAT instrument and were found to be in good agreement^22^. Here, we study the enhancements in methane observed over two oil and gas production regions in the U.S., the Uintah basin in Utah and the Permian Basin on the border of Texas and New Mexico. Using corroborating evidence from ground-based and airborne measurements, we show that the observed methane enhancements in single overpasses can be attributed to emissions into the boundary layer from oil and natural gas production. These results suggest that the future determination of regional methane emissions with a high time resolution and soon after the time of emission will be feasible.

## Results

### TROPOMI observations

**Figure 1:**
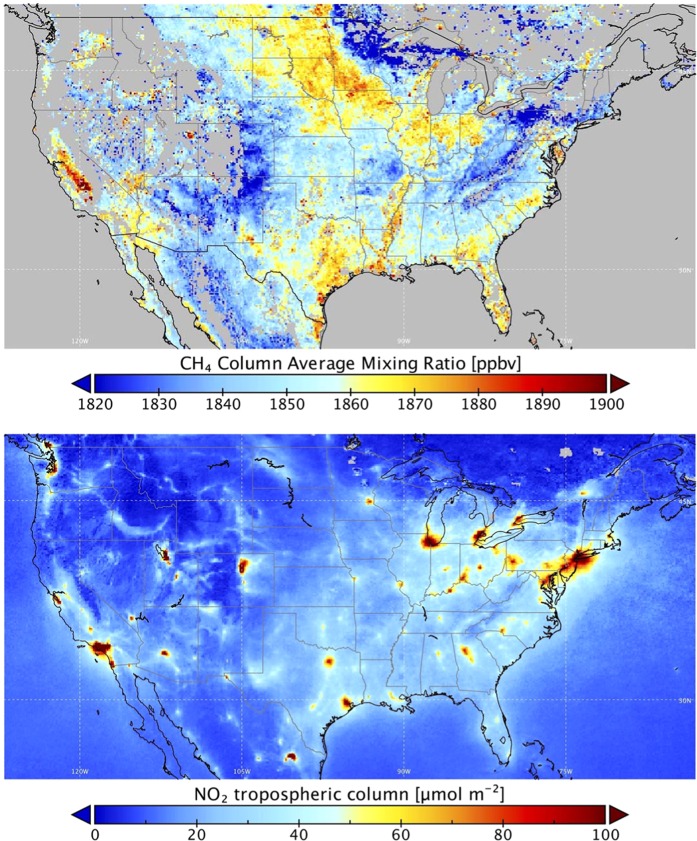
Average TROPOMI columns for (top) methane and (bottom) tropospheric NO_2_ over the contiguous United States between 1 December 2018 and 31 March 2019. Retrieved columns have been binned on a 0.1° × 0.125° latitude-longitude grid for this analysis.

Average methane columns over the contiguous U.S. between 1 December 2018 and 31 March 2019 are shown in Fig. 1. The number of TROPOMI retrievals per grid box during this period is shown in Fig. S1 and was limited over some regions due to cloud cover, low surface albedo, topography and other factors that affect the data quality. Enhancements in methane columns are for example observed:

- Over the Central Valley of California, a region with strong methane emissions from agriculture (both livestock and rice cultivation) and from oil and natural gas production. The enhancements were particularly clear in December 2018 and January 2019, as a result of wintertime inversions that trap emitted methane in the boundary layer.
- In the Uintah basin in Utah in the northeastern corner of the state. This is a region where strong inversions can trap emissions from oil and gas production in the winter. Accordingly, the enhancements were particularly clear in December 2018.
- Over Texas, which has several oil and gas producing regions. Both oil and gas are produced in the Permian Basin in the Southeast corner of New Mexico and the adjacent parts of Texas, and in the Eagle Ford south of San Antonio. Natural gas production also takes place in the Barnett Shale west of Dallas - Fort Worth, and in the Haynesville on the border of Texas and Louisiana.
- Over several states with intense agricultural production of corn and soybean^23^ ranging from North Dakota through Minnesota, Illinois and Ohio as well as the Mississippi Delta region. Rather than an actual emission of methane, we suspect that retrieval biases due to low surface albedo in the winter, when the agricultural soils are bare, may play a role and need to be further investigated.
- Over Florida, which may be related to emissions from wetlands.

The San Juan basin in the northwestern part of New Mexico does not show up consistently in the TROPOMI data so far, although enhancements at the northern end of the basin were observed on two days in January 2019 (Fig. S2). A previous study identified a hot spot in methane in this basin using data from SCIAMACHY^16^.

Average tropospheric NO_2_ columns between 1 December 2018 and 31 March 2019 are also shown in Fig. 1. In this case, the strongest enhancements are observed over metropolitan areas such as New York City, Chicago, Denver, Houston, Los Angeles and many others. Enhanced NO_2_ columns are also observed over oil and gas producing regions, most clearly over the Permian Basin and Eagle Ford in Texas, and the Bakken in North Dakota. Enhanced NO_2_ over these regions was previously identified in the OMI data^24^.

The methane columns shown in Fig. 1 have not been validated in detail. A comparison with GOSAT data was previously published^22^ and a comparison with the measurements by the Total Carbon Column Observing Network (TCCON) is currently in progress. We include here a comparison between TROPOMI measurements that are co-located with the Lamont TCCON site in Oklahoma within a 300-km radius (Fig. S3). The dynamic range in methane columns over the comparison period was limited, but the two data sets both show periods with enhanced columns in August 2018 and in October-December 2018, and were correlated with a linear correlation coefficient, r, of 0.6. The average bias between the TROPOMI and Lamont TCCON data sets was −7.5 ± 9.9 ppbv, where the error represents the 1-σ variability in the bias from individual days.

The accuracy of the TROPOMI methane column product for low surface albedo needs further investigation and so data included in this paper are all limited to a surface albedo higher than 0.05, except for Fig. S2 where an albedo threshold of 0.03 was applied. Methane column mixing ratios over the Great Lakes region and into Canada are occasionally lower than the surface values and possibly affected by low tropopause heights and stratospheric air masses with reduced methane. Nevertheless, the column enhancements over Utah and Texas appear to be readily attributable to emissions associated with oil and gas production, and this hypothesis is evaluated in the next two sections.

### Uintah basin

The Uintah Basin in Utah is a smaller natural gas and, to a lesser extent, oil producing region in the northeastern part of Utah. Drilling activity has declined since 2008 and natural gas production is currently decreasing (Fig. S4). The basin is surrounded by mountains and atmospheric studies in the region were sparked by the occurrence of winter ozone formation^25,26^. This unusual phenomenon occurs when emissions from oil and gas production accumulate under cold-pool conditions strengthened by a snow cover, and react photochemically over the course of days to build up ozone to levels that far exceed other polluted regions of the U.S.

**Figure 2:**
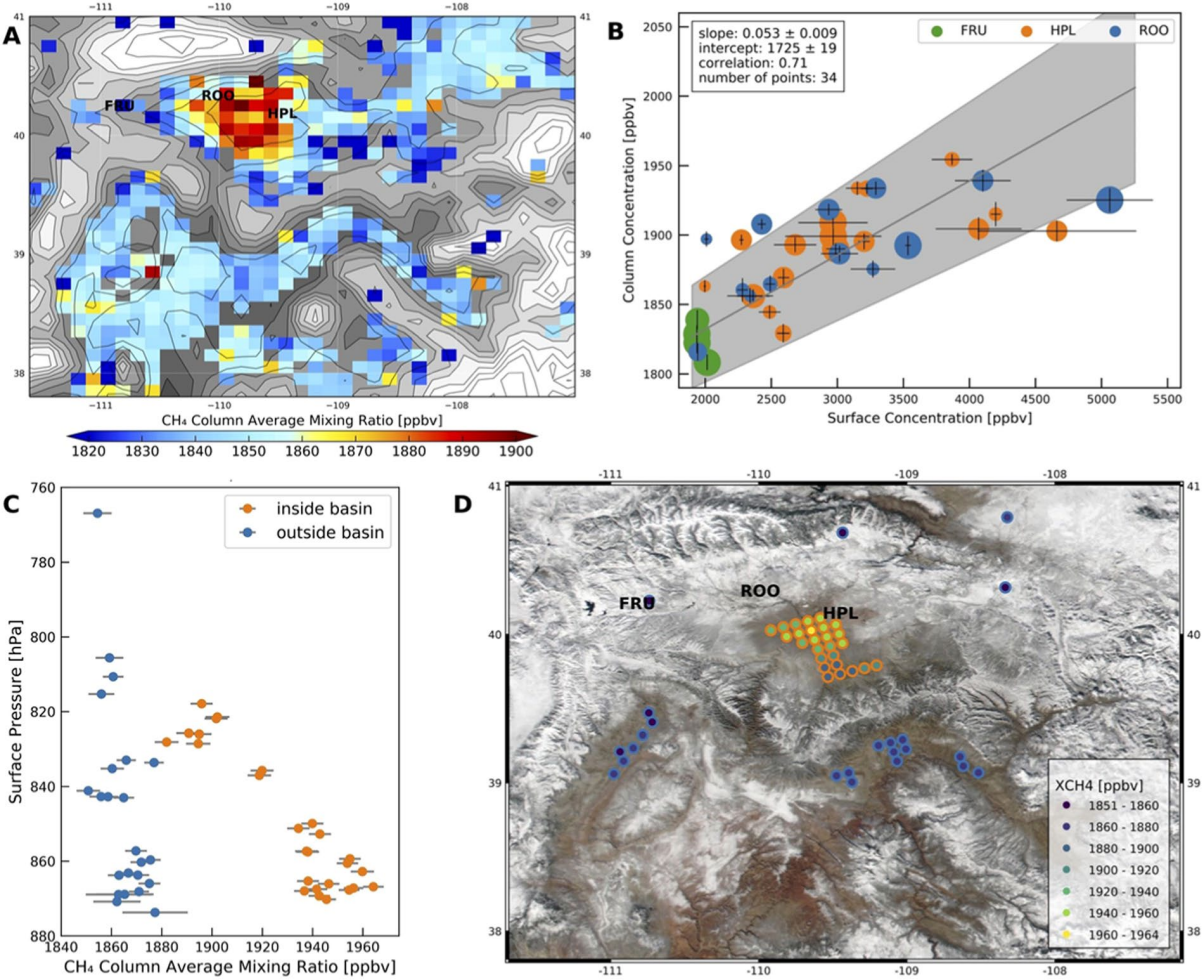
TROPOMI methane observations over the Uintah Basin in Utah. (**A**) Average methane columns from 1 December 2018 through 31 March 2019. The grey shaded background shows the surface elevation from the GMTED 0.125° digital elevation map. The contour lines indicate 50-meter steps in the surface elevation. (**B**) Comparison between the TROPOMI methane columns with the coincident *in-situ* measurements at Horsepool (HPL), Roosevelt (ROO) and Fruitland (FRU). The size of the symbols indicates the distance between the satellite ground pixel and the surface monitor, where large symbols indicate better collocation. Error bars in surface concentrations represent the standard deviation in the 60-min mean centered on the TROPOMI overpass time. Error bars in the column concentration represent 2 × the reported precision of the retrieval as recommended. (**C**) Methane columns on 9 December 2018 as a function of the surface pressure, for ground pixels inside the Uintah basin (orange) and outside the basin (blue). (**D**) Location of the methane column measurements shown in panel C, where the inside of the symbols indicates the CH_4_ mixing ratio and the outside is orange for pixels inside the Uintah basin, and blue outside the basin. Methane is not retrieved over pixels with too much variability in the terrain elevation, which explains the many missing pixels in panel A and D.

The TROPOMI methane measurements show a clear hotspot over the Uintah basin (Fig. 2A). Enhanced methane columns were observed on the south side and deeper part of the basin, where most of the oil and natural gas wells are situated^11^. Data gaps in the TROPOMI data are caused by clouds, topographic features and low surface albedo. The methane hotspot in the Uintah Basin became less obvious from December 2018 through March 2019 as the cold-pool conditions that trap emissions in the basin became less prevalent (Fig. S5). NO_2_ columns over the Uintah Basin are strongly affected by emissions from the Bonanza power plant^26^ and are not included in this analysis.

Methane has been measured *in-situ* in the basin since 2015 at three different locations indicated in Fig. 2A. The Horsepool and Roosevelt monitors are situated at lower elevation in the basin and are frequently exposed to high methane in the winter when emissions are trapped under cold-pool conditions. The Fruitland monitor is situated at a higher elevation and does not see similarly high methane enhancements^27^. Figure 2B compares the TROPOMI methane columns between December 2018 and March 2019 with data from the three monitors. The *in-situ* measurements were averaged over a 60-min interval centered on the overpass time of the TROPOMI instrument. TROPOMI data were limited to those pixels that were within 30 km of the monitor, with the size of the symbol representing the proximity. Data were also selected for surface albedos >0.05. The column average methane mixing ratios from TROPOMI correlate with the *in-situ* measurements with a correlation coefficient of 0.71. The effect of selecting for surface albedo is shown in Fig. S6. A less strict criterion results in more outliers, but the correlation between the column and *in-situ* measurements remains.

Figure 2B shows a linear regression slope between the column-averaged and surface methane mixing ratios of 0.053. This value is much lower than one, because methane is only enhanced in a shallow boundary layer. Assuming a tropopause altitude of ~10 km and a surface altitude of 1.5 km, this implies that methane accumulates in a layer of ~450 m thick (0.053 × 8500 m). This layer height is in the range of previous observations in the basin. During cold-pool conditions, the height of the polluted layer is lower initially (200–300 m)^26^ but can grow as cold-pool conditions persist and emissions gradually mix to higher elevations. Deeper boundary layers of more than ~1500 m were also observed in the winter in the absence of cold-pool conditions^11^. More insight into the altitude distribution of the methane is obtained by plotting the column average mixing ratios of methane observed on a single day, 9 December 2018, as a function of the surface pressure, i.e. elevation (Fig. 2C), with the location of the columns shown in Fig. 2D. In the absence of local emissions, the column-averaged methane mixing ratios do depend slightly on surface elevation, which may explain the observations outside the basin, but the much stronger altitude dependence inside the basin can only be attributed to the build-up of methane in a shallow layer. This analysis also suggests that the depth of the layer with enhanced methane is ~50 hPa or ~500 m. It should be noted that the Uintah basin was snow-free on 9 December 2018 (Fig. 2D), so the inversion may not have been strong and the mixed layer deeper than during cold-pool conditions^28^.

We conclude that the TROPOMI hotspot observed over the Uintah Basin is caused by enhanced methane in the boundary layer in agreement with surface monitors. Continued surface and TROPOMI measurements over the Uintah Basin will be useful to further develop and evaluate emissions estimates under cold-pool conditions^27^, which can then be applied with more confidence for other, similar basins.

### Permian basin

The Permian Basin in Texas and New Mexico has been a very large production region for both oil and gas in the United States for many years (Fig. S4). The region has seen increases in extraction of both oil and natural gas by unconventional means since 2010, i.e. horizontal drilling and hydraulic fracturing that allow extraction from shale and tight sands formations. Unlike the Bakken in North Dakota and the Eagle Ford in Texas, the Permian Basin did not see a decrease in oil production after the 2015 downturn in drilling and is currently developing very rapidly.

**Figure 3:**
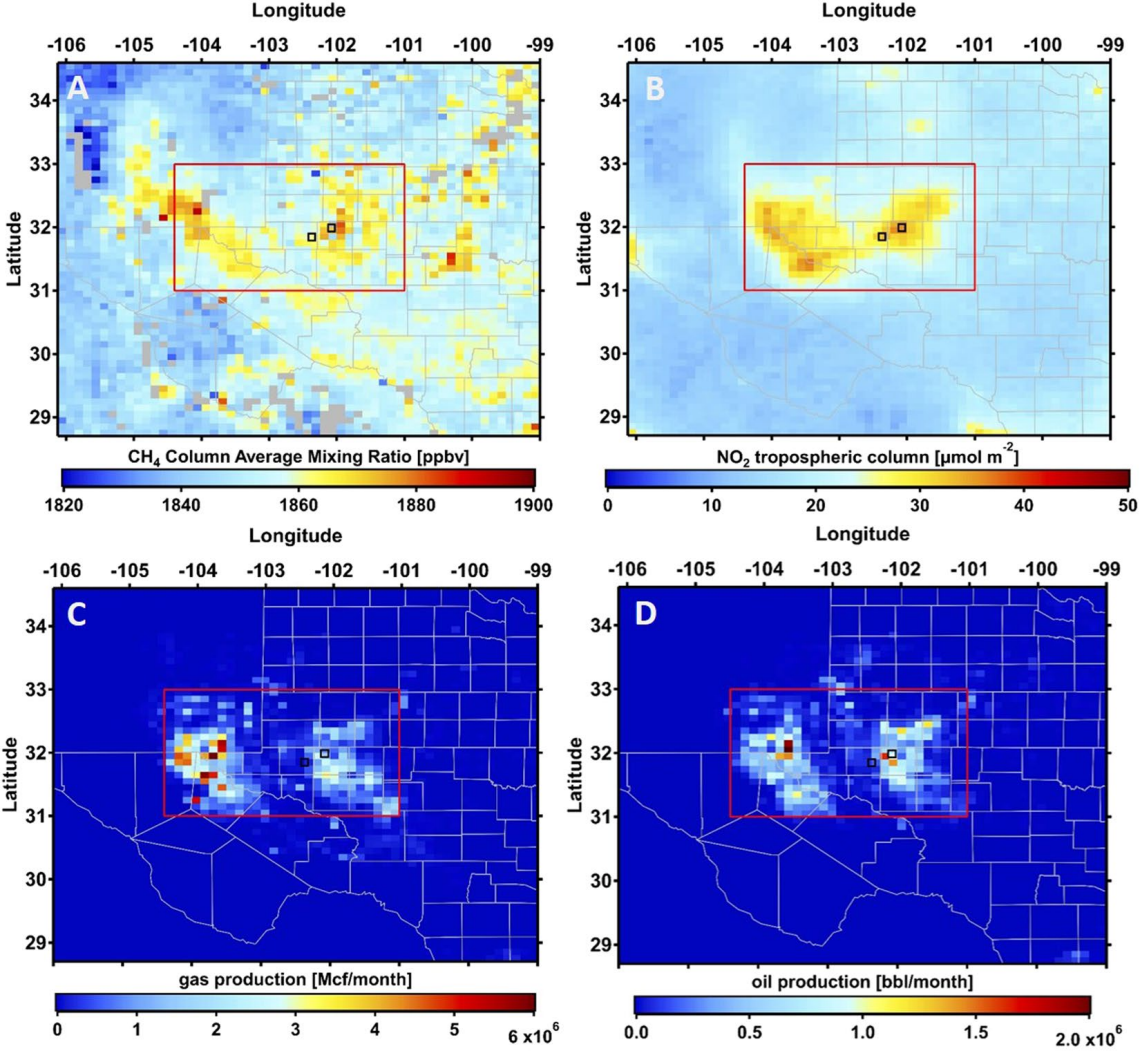
(**A**) TROPOMI methane and (**B**) tropospheric NO_2_ over the Permian basin averaged from 1 December 2018 through 31 August 2019. Panels C and D show the natural gas and oil production averaged over that same period. The locations of the largest cities in the region, Midland and Odessa, are indicated by the open squares in all 4 panels.

**Figure 4:**
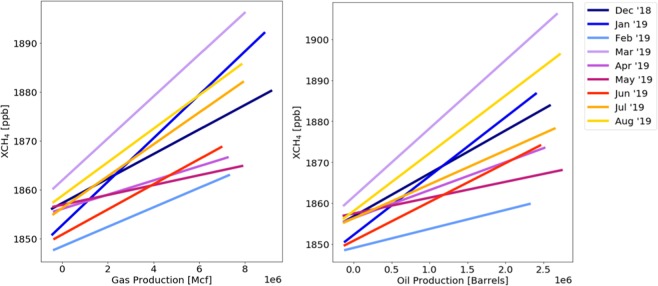
Average relationships between monthly averaged, gridded methane columns vs. natural gas (left) and vs. oil production (right) over the Permian Basin.

Figure 3 shows the methane and tropospheric NO_2_ columns over the Permian basin averaged over the 1 December 2018 through 31 August 2019 period. Natural gas and oil production volumes are shown in Fig. 3C,D. Both methane and NO_2_ show enhancements over the Delaware basin in the west, and the Midland basin in the eastern part of the Permian Basin. NO_2_ is also enhanced over Midland and Odessa, the two main cities in the region, but NO_x_ emissions in these cities are clearly not the only source. Figure 4 illustrates the relationship between methane and oil and gas production in more detail. For each month between December 2018 and August 2019, we regressed gridded methane columns vs. gridded natural gas and oil production, and the best fits for each month are shown in Fig. 4. Examples of the individual fits are shown for August 2019 in Fig. S7. It is seen that pixels with higher production are associated with significantly higher methane columns, and that the slopes are similar between different months.

Using airborne measurements, it has been shown that methane and NO_x_ emissions in oil and gas production regions are correlated^29^. While these two trace gases are not from the exact same sources, the emissions are collocated thereby causing their correlation in the atmosphere. For example, methane is released from the wellhead, separator and compressor on each well site, while NO_x_ is released from internal combustion engines that are used to run this equipment^30^. All these parts of the infrastructure are sufficiently close to each other that their emissions appear to come from the same location when sampling at a distance. Also, while the emissions from each well site can be different, an average ΔNO_x_/ΔCH_4_ ratio is observed when averaging over 100 s of sites. Here, ΔNO_x_ and ΔCH_4_ refer to their enhancements over the background and are determined from the linear regression between their mixing ratios, with the background in methane signifying the background mixing ratio. During airborne measurements in 2015 in the Permian Basin, this ratio was observed to be (9.0 ± 0.2) × 10^−3^ ^29^.

**Figure 5:**
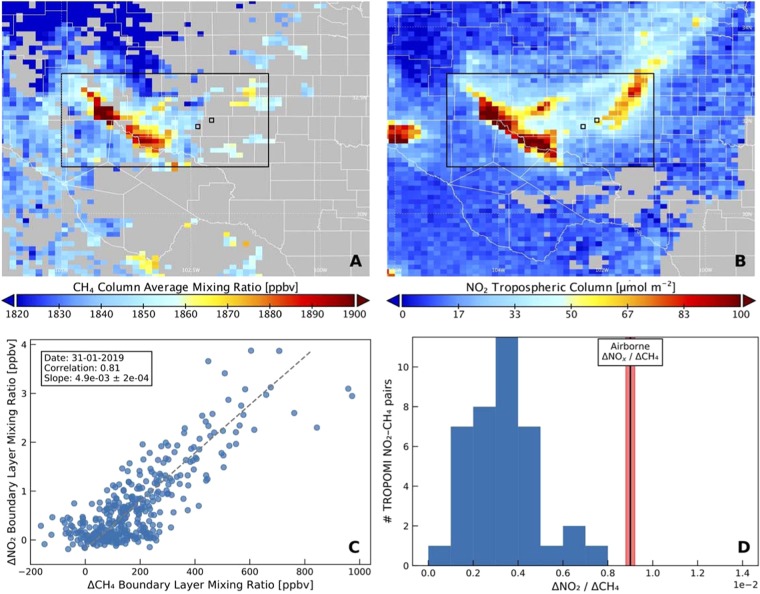
Correlation between TROPOMI methane and NO_2_ columns in the Permian Basin. Columns for (**A**) methane and (**B**) tropospheric NO_2_ data from 31 January 2019 show enhancements in the same areas. (**C**) Scatter plot of NO_2_ vs. methane boundary layer mixing ratios calculated from the TROPOMI columns in the Permian Basin box highlighted by the thick black line; the NO_2_ enhancement west of the box is due to El Paso-Ciudad Juarez. NO_2_ and methane were correlated on 31 January with a linear correlation coefficient of 0.81 and an NO_2_-to-CH_4_ slope of (4.9 ± 0.2) × 10^−3^. (**D**) A histogram of NO_2_-to-CH_4_ slopes from all overpasses with r > 0.4 between 1 December 2018 and 31 March 2019 shows a distribution that is limited at the high end by the NO_2_-to-CH_4_ slope of (9.0 ± 0.2) × 10^−3^ observed from airborne measurements indicated by the line and the red area.

The TROPOMI methane and NO_2_ columns in the Permian Basin were often found to be correlated on individual days. Examples are shown for 31 January 2019 in Fig. 5 and for 25 January 2019 in Fig. S8. Within a box that encompasses the Permian Basin indicated with the thick line, NO_2_ and methane correlated with a correlation coefficient of 0.81 on 31 January (Fig. 4C) and of 0.86 on 25 January (Fig. S8). No concurrent enhancements were seen for the aerosol optical thickness derived from the NPP VIIRS measurements, which were obtained within minutes from the TROPOMI overpass, ruling out an aerosol interference. In the December 2018–March 2019 period, NO_2_ and methane were correlated with r > 0.4 on 45 out of 55 days with available data.

The relation between the NO_2_ and CH_4_ in the boundary layer is assessed for each day for which more than 10 NO_2_-CH_4_ data pairs are available. First, we subtract the background for both NO_2_ and CH_4_. The background is estimated as the 10th percentile of the values. Next, we convert the NO_2_ column and CH_4_ mixing ratios into boundary layer mixing ratios, by assuming a boundary layer thickness of 100 hPa. For NO_2_ this is done by converting the number concentration into a mixing ratio. For CH_4_ we rescale the column mixing ratio by the surface pressure divided by 100 hPa. We fit a linear relationship through the NO_2_-CH_4_ data points, using an orthogonal least-squares fit. Before applying this fit, we apply a scaling such that both NO_2_ and CH_4_ are in the range 0–1. On 31 January, the ΔNO_2_/ΔCH_4_ ratio was found to be (4.9 ± 0.2) × 10^−3^. The distribution in the ΔNO_2_/ΔCH_4_ ratios from all overpasses between 1 December 2018 and 31 March 2019 with r > 0.4 is shown in Fig. 5D. Also added to this graph is the ΔNO_x_/ΔCH_4_ ratio of (9.0 ± 0.2) × 10^−3^ reported from airborne measurements in the Permian Basin^29^. This ratio is at the high end of the ΔNO_2_/ΔCH_4_ ratios from TROPOMI, which is entirely reasonable given that NO_x_ includes NO in addition to NO_2_, and because of photochemical removal, which can remove a fraction of NO_x_ on the timescale of atmospheric transport across the basin.

We conclude that the TROPOMI methane enhancements observed over the Permian Basin are caused by emissions in the boundary layer that include those from oil and gas production. The TROPOMI data show separate enhancements over the eastern (Midland) and western (Delaware) parts of the Permian Basin, which can be explored in more detail when more data are available.

## Discussion

Using methane and NO_2_ column measurements from the new TROPOMI instrument, we have shown that emissions from oil and gas production in the Uintah and Permian Basins can be observed in the data from individual overpasses. This is a vast improvement over measurements from previous satellite instruments, which typically needed to be averaged over a year or more to quantify trends and regional enhancements in methane emissions.

In the future, TROPOMI will likely allow the study of trends in methane and NO_x_ emissions from oil and gas production. It will be of interest to look for the correlations between, on one hand, methane and NO_2_ columns with, on the other hand, proxies for industrial activity such as active drill rig counts, production volumes of oil, gas and condensate, and natural gas flare data. Such analyses may allow the relative importance of different industrial activities to be separated. The current analysis also needs to be extended to other oil and production regions in North America once more data are available, and to production regions outside of North America.

The TROPOMI data will likely also allow the quantification of methane and NO_2_ emissions through inverse modeling. For methane, this can possibly be done directly, although the accurate modeling of tropopause height, stratospheric removal and boundary conditions of the modeling domain are all significant challenges^31^. An indirect way of determining methane emissions may be through the inverse modeling of NO_x_ emissions^32,33^ and the scaling to methane through observed NO_2_/CH_4_ ratios.

Finally, *in-situ* measurements in oil and gas production regions and coordinated airborne measurements would allow further verification of the results obtained in this analysis and extension to other regions.

## Methods

### TROPOMI satellite instrument

TROPOMI is the single payload on board of the EU Copernicus Sentinel-5 Precursor satellite, which was launched in October 2017 into a Sun-synchronous orbit with an overpass time of 13:30 local solar time^21^. TROPOMI is a push-broom imaging spectrometer with a swath width of 2,600 km and a ground pixel of 3.5–7 × 7 km^2^ at the sub-satellite point. TROPOMI has two spectrometer modules, the first covering bands in the ultraviolet, visible, and near-infrared (NIR) and the second dedicated to the shortwave infrared (SWIR) band centered around 2.3 μm.

### TROPOMI methane retrieval

We use the TROPOMI operational CH_4,_ data product (Hu 2016) version 1.2 (10.5270/S5P-3p6lnwd), which implements the RemoTec retrieval algorithm^34,35^. The operational algorithm uses a two-band retrieval approach using the NIR and SWIR bands. The SWIR band contains the CH_4_ information and the NIR band is used for correction for aerosols. From the operational data we use bias-corrected column average mixing ratios, with a quality value larger than 0.5, which filters the data for low data quality, high solar and viewing zenith angles and high aerosol loading. We estimate the uncertainty in the column average CH_4_ mixing ratios by multiplying the reported retrieval precision with a factor of 2, as recommended by the data providers.

For the maps shown in Fig. 1, and for the comparisons between NO_2_ and CH_4_ we constructed daily data on a 0.1° × 0.125° latitude-longitude grid. For the CH_4_ data, only retrievals with a surface albedo larger than 0.05 are included in the gridded data.

### TROPOMI NO_2_ retrieval

We use the TROPOMI operational NO_2_ data product version 1.2 (10.5270/S5P-s4ljg54). The NO_2_ processing system uses a DOAS (Differential Optical Absorption Spectroscopy) fit on the visible spectra. The tropospheric NO_2_ column is determined by subtracting the stratospheric NO_2_ column amount and corrections for the effects of surface reflectance and clouds^36^. We use the NO_2_ retrievals with a quality value larger than 0.75.

### *In-situ* measurements in the uintah basin

*In-situ* measurements of CH_4_ (along with CO_2_ and H_2_O) at three sites in the Uintah Basin have been carried out by the University of Utah since 2015 using Los Gatos Ultra-Portable Greenhouse Gas Analyzers (Los Gatos Research Inc., San Jose, CA). The analyzers take data every 10 seconds and are calibrated every three hours with reference gas cylinders traceable to the World Meteorological Organization scale. For more details regarding the measurement setup and instrument uncertainties, we refer the reader to Foster *et al*.^10^ and Bares *et al*.^37^.

### TCCON measurements at lamont

Measurements from the Total Carbon Column Observing Network (TCCON) at Lamont, Oklahoma (36.604 N, 97.486 W) are used to evaluate TROPOMI data for a location that is closest to the region of interest (Fig. S3). TCCON consists of a network of ground-based Fourier-Transform Infrared Spectrometers that record spectra of the sun and allow accurate and precise retrievals of column-averaged abundances of atmospheric constituents including CH_4_. Data are available at 10.14291/tccon.ggg2014.lamont01.R1/1255070 ^38^.

### Permian basin production data

Oil and natural gas production data were obtained at the well level using the Drillinginfo tool provided by Enverus and were averaged over the same 0.1° × 0.125° latitude-longitude grid boxes as used for the methane columns. Total production volumes of oil and natural gas for the U.S. and for the Permian Basin were also obtained from the U.S. Energy Information Administration (www.eia.gov) and for Utah from the Utah Division of Oil, Gas and Mining (oilgas.ogm.utah.gov) summing the data for the Uintah and Duchesne counties. Drill rig counts are from Baker Hughes (bakerhughes.com) with only statewide data available for Utah.

## Supplementary information

Supplementary Information.

## Data Availability

TROPOMI methane (10.5270/S5P-3p6lnwd) and NO_2_ data (10.5270/S5P-s4ljg54) are publicly available. The *in-situ* methane measurements in the Uintah Basin are available at https://air.utah.edu and the raw data are provided upon request by J.C. Lin. The TCCON data are publicly available at 10.14291/tccon.ggg2014.lamont01.R1/1255070.
