# Supplementary material for: Daily Satellite Observations of Methane from Oil and Gas Production Regions in the United States
Source: Sci Rep. 2020 Jan 28;10:1379. doi: 10.1038/s41598-020-57678-4 (PMC6987228; doi:10.1038/s41598-020-57678-4)

**Supplementary Information**

**Daily Satellite Observations of Methane from Oil and Gas Production Regions
in the United States**

Joost A. de Gouw^1,2,*♦^, J. Pepijn Veefkind^3,4♦^, Esther Roosenbrand^1,4^, Barbara Dix^1^,
John C. Lin^5^, Jochen Landgraf^6^, and Pieternel F. Levelt^3,4^

^1^ Cooperative Institute for Research in Environmental Sciences, University of Colorado, Boulder, CO, United States

^2^ Department of Chemistry, University of Colorado, Boulder, CO, United States

^3^ Royal Netherlands Meteorological Institute, de Bilt, the Netherlands

^4^ Faculty of Civil Engineering and Geosciences, Delft University of Technology, Delft, the Netherlands

^5^ Department of Atmospheric Sciences, University of Utah, Salt Lake City, UT, United States

^6^ SRON Netherlands Institute for Space Research, Utrecht, the Netherlands

^*^ Corresponding Author

^♦^ These Authors contributed equally to this work

**Figure S1:** Number of TROPOMI ground pixels per grid box for the average methane columns shown in Figure 1.


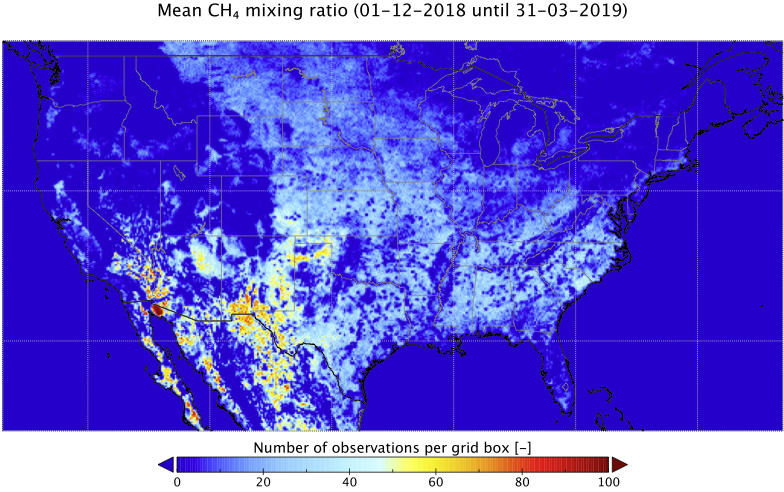


**Figure S2:** Methane column enhancements over the San Juan basin as observed from TROPOMI. A previous study had identified a methane hotspot in this region using data from the SCIAMACHY satellite instrument. So far, TROPOMI has only detected methane enhancements on 3 January (left panel) and 4 January 2019 (right panel). In these panels, only retrieved methane columns with a surface albedo larger than 0.03 are shown.


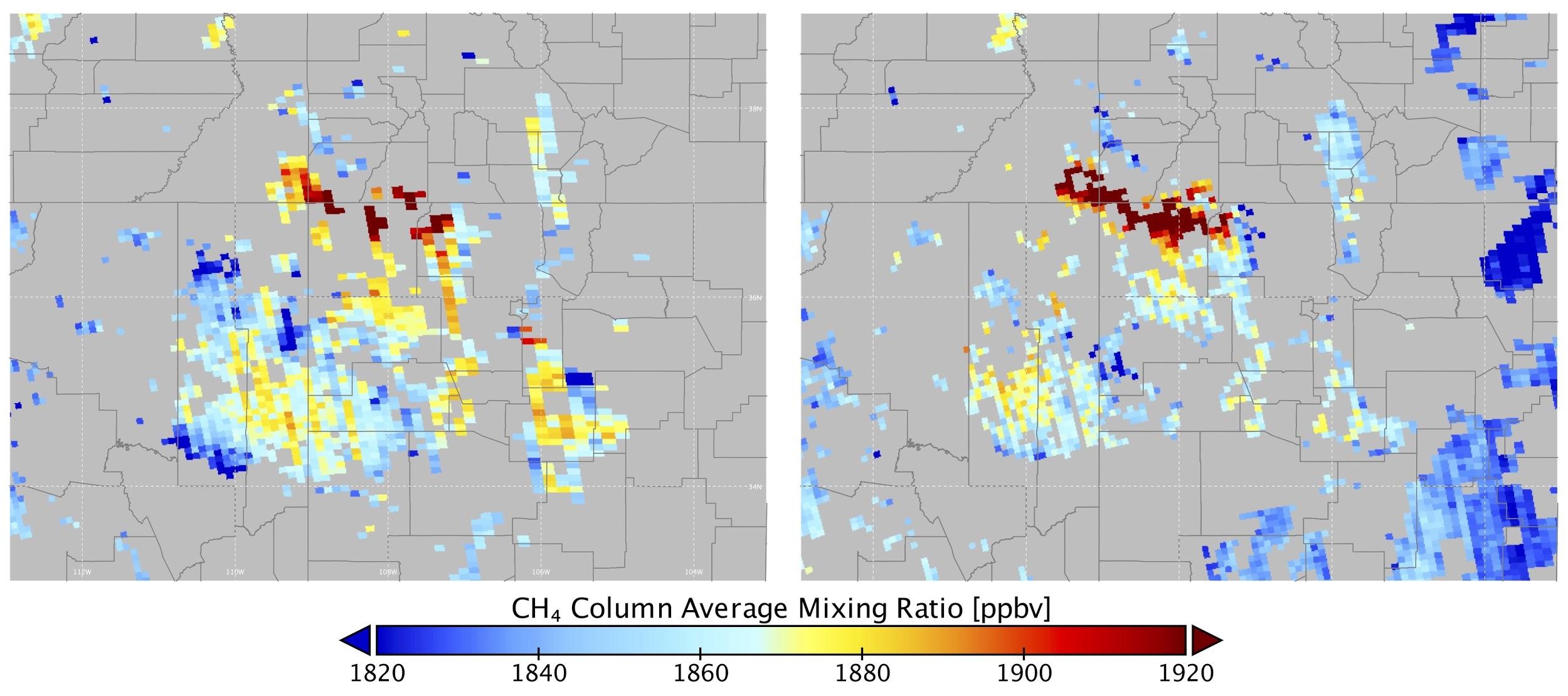


**Figure S3:** Comparison between column average methane mixing ratios from the TCCON site at Lamont in Oklahoma with the TROPOMI data obtained within a 300-km radius around the site. The two data sets correlate with a linear correlation coefficient of 0.6. The average bias between the TROPOMI and Lamont TCCON data sets was -7.5±9.9 ppbv, where the error represents the 1-σ variability in the bias from individual days.


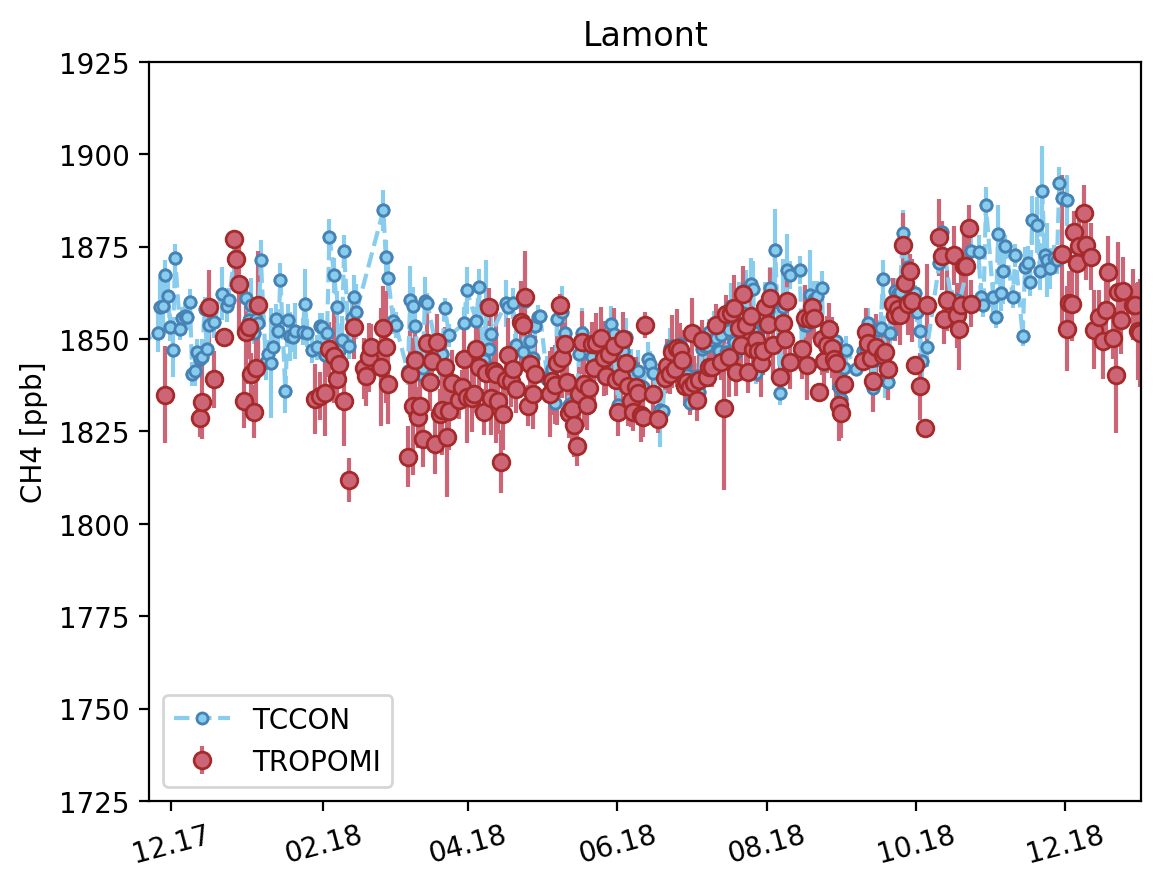


**Figure S4:** Summary of (A) drilling activity including the total and the fraction of wells that were drilled horizontally, (B) gas production, and (C) oil production in the U.S., and in the Permian and Uintah Basins. Production numbers for the U.S. and Permian are obtained from the U.S. Energy Information Administration ([eia.gov](https://www.eia.gov/)), production data for the Uintah Basin are from the Utah Division of Oil, Gas and Mining ([oilgas.ogm.utah.gov](https://www.ogm.utah.gov/); combining data for the Uintah and Duchesne counties), active drill rig counts are from Baker Hughes ([bakerhughes.com](https://www.bhge.com/); only statewide data available for Utah).


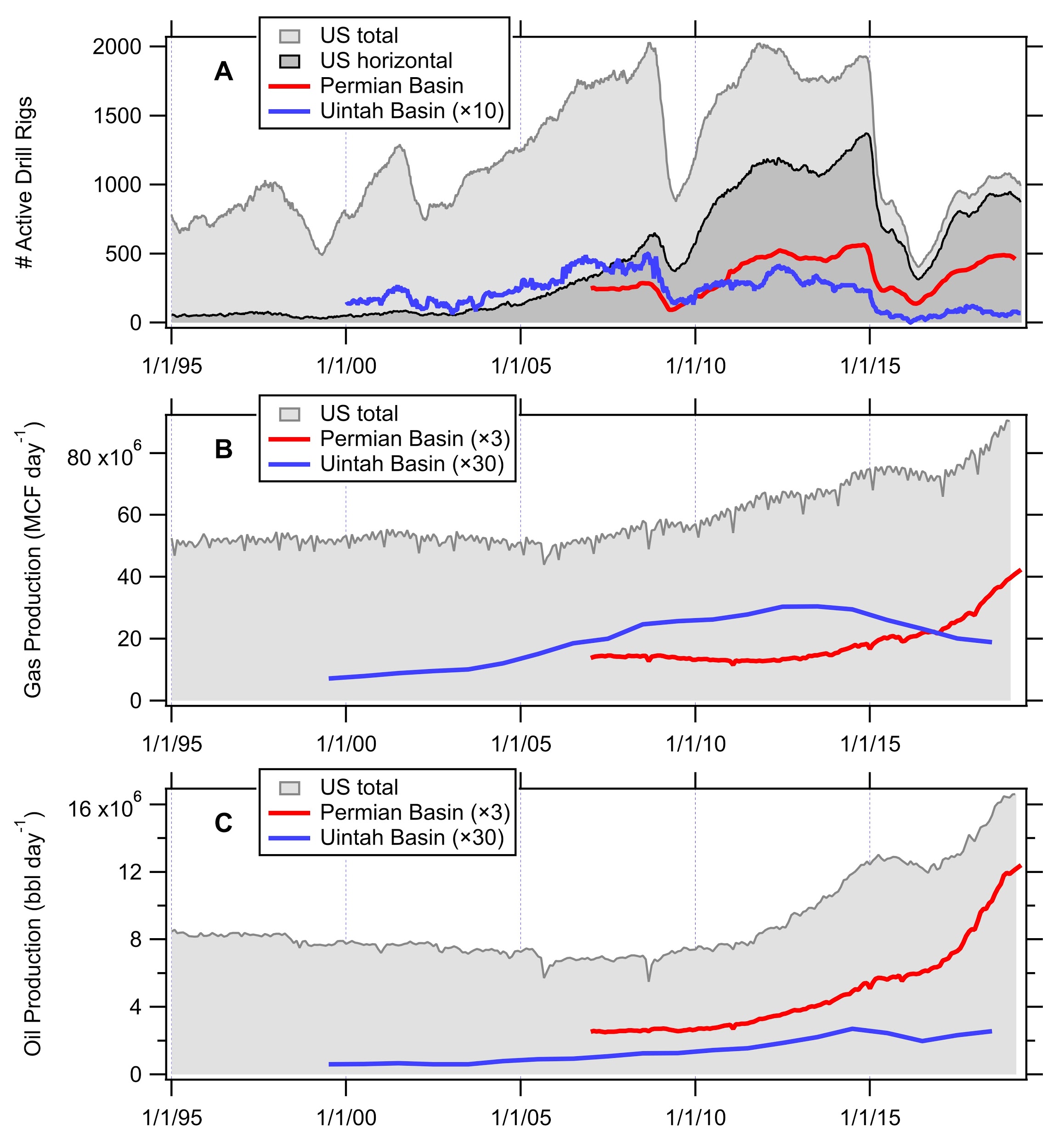


**Figure S5:** Average methane columns over the Uintah basin and surrounding areas in Northwest Utah for (A) December 2018, (B) January 2019, (C) February 2019 and (D) March 2019. The grayscale background shows the surface elevation.


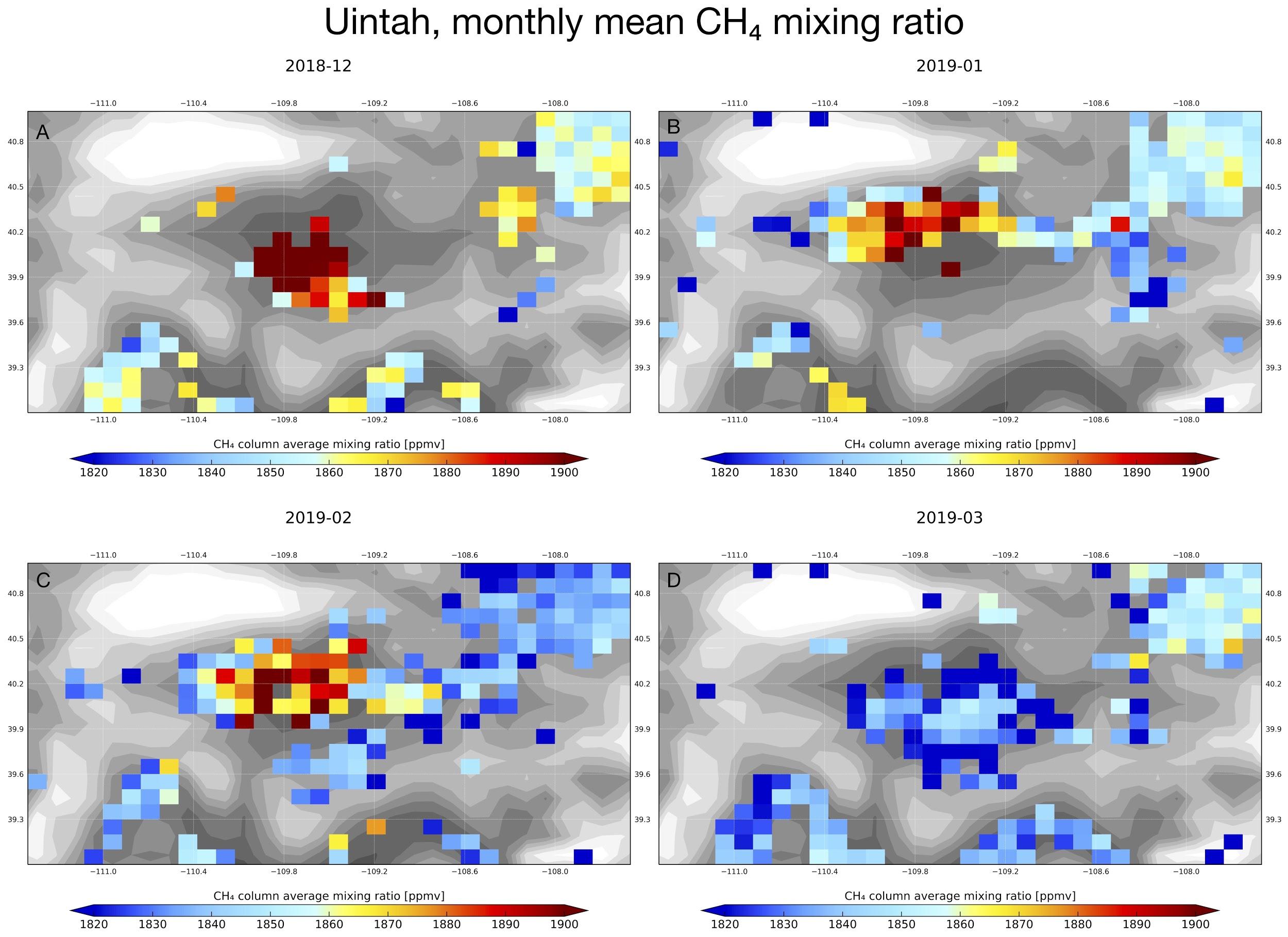


**Figure S6:** Comparison between the TROPOMI methane columns over the Uintah basin and the in-situ measurements made at Horsepool (HPL), Roosevelt (ROO) and Fruitland (FRU). The left panel compares all TROPOMI data within 30 km from the in-situ monitor, with the size of the symbol representing the distance (larger is closer). The right panel contains all TROPOMI data within 30 km, but further constrained to a surface albedo of >0.05. Selecting for surface albedo improves the correlation coefficient from 0.62 to 0.71. The grey areas in both panels show the errors in the linear regression taking into account the uncertainties in both the TROPOMI columns and the variability in surface measurements.


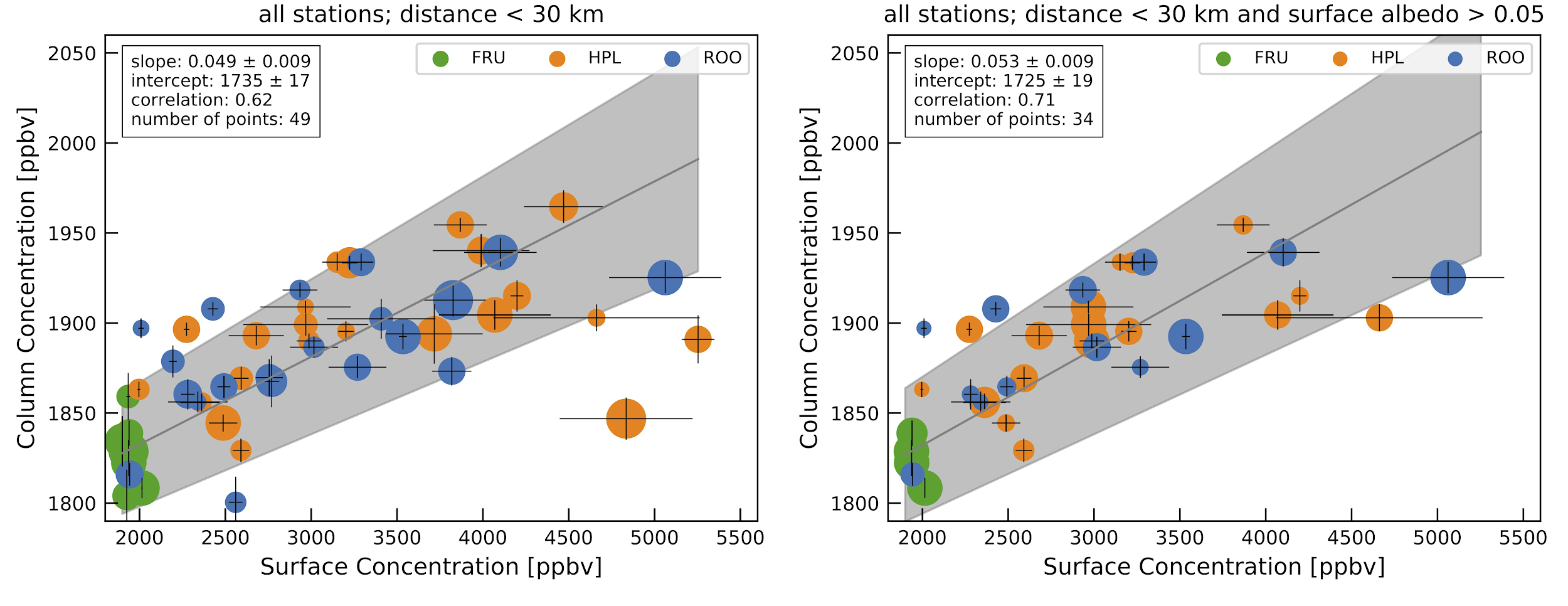


**Figure S7:** Correlation between TROPOMI methane and (top) natural gas production, and (bottom) oil production in the Permian Basin. Data are for August 2019. Blue symbols show the data for each pixel in the Permian Basin. The blue line and shaded region represent the best fit to the data and the uncertainty in the fit. The orange symbols and uncertainty ranges represent the average methane columns, and the uncertainties therein, in the binned production ranges indicated by the dotted vertical lines.

**
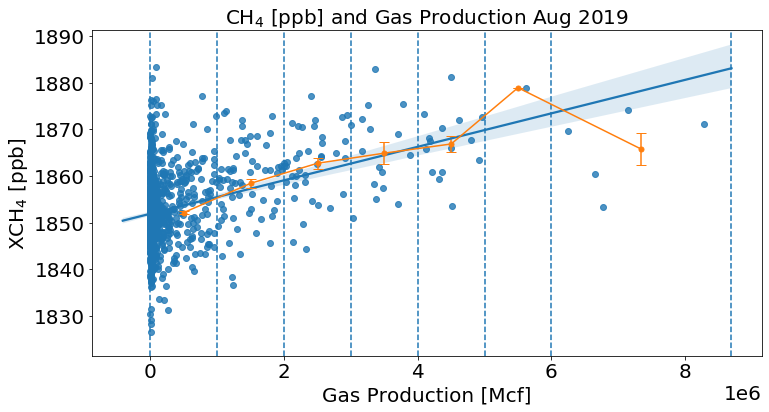
**

**
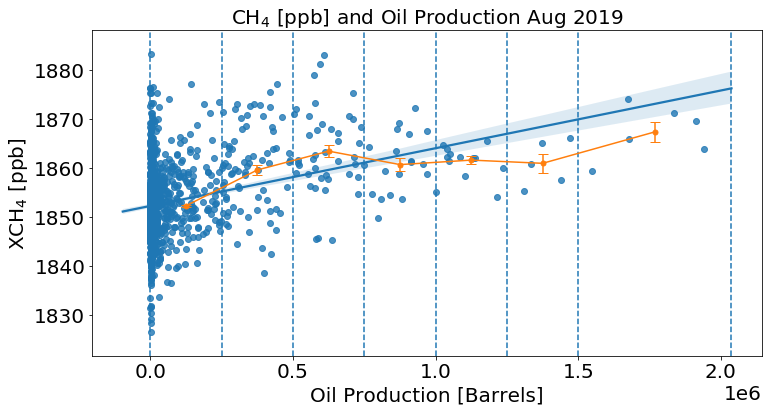
**

**Figure S8:** Correlation between TROPOMI methane and NO_2_ columns in the Permian Basin. Columns for (A) methane and (B) tropospheric NO_2_ data from 25 January 2019 show enhancements in the same areas. These graphs are similar to Figure 4B-C, but for a different day. NO_2_ and methane were correlated on 25 January with a linear correlation coefficient of 0.86 and an NO_2_-to-CH_4_ slope of 0.0040 ± 0.0001. A histogram of NO_2_-to-CH_4_ slopes from all overpasses with r>0.4 is shown in Figure 4D.


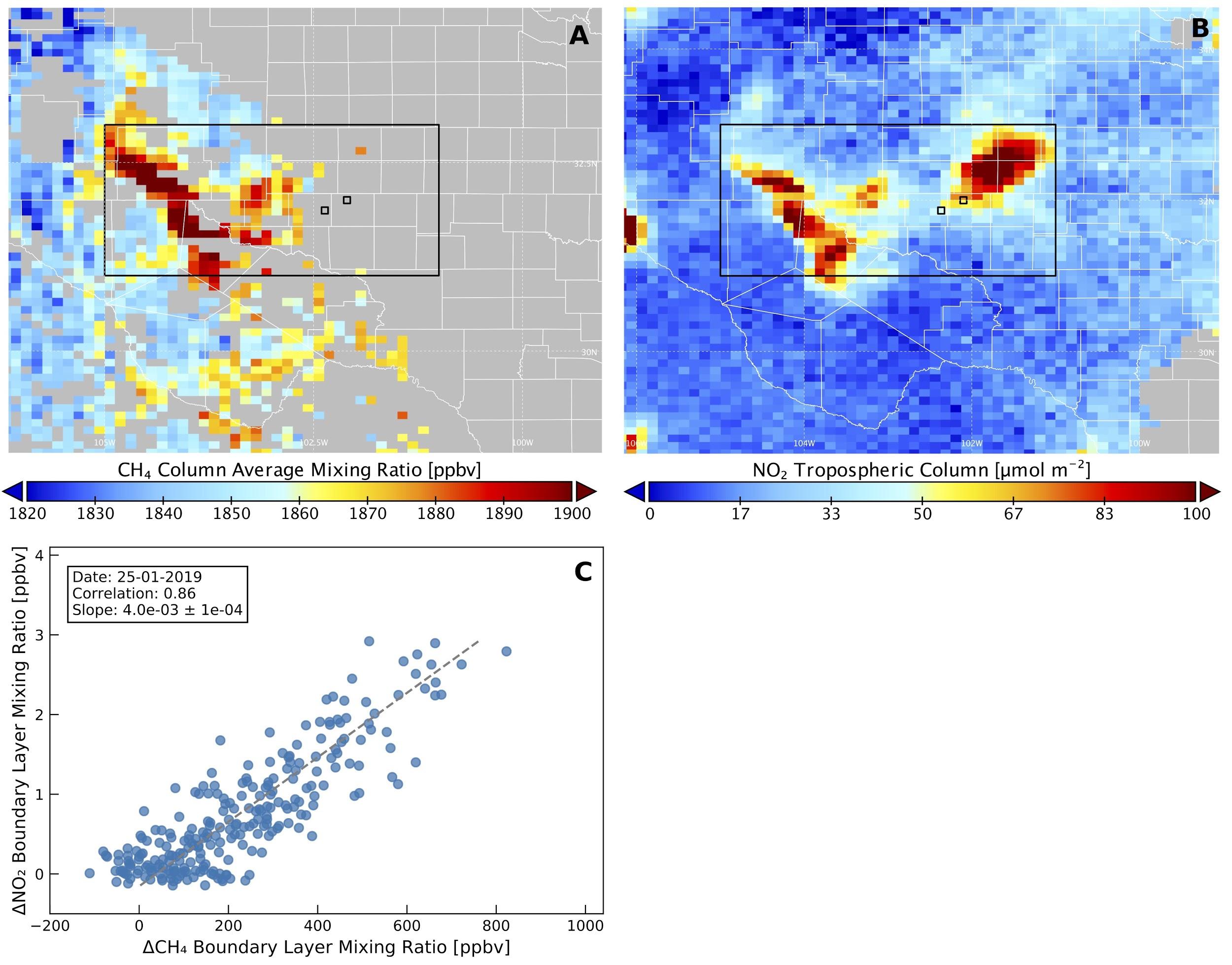

Supplement: Supplementary file 1 — Supplementary Information. [file 41598_2020_57678_MOESM1_ESM.docx]
